# Anticancer Activity of *Anthopleura anjunae* Oligopeptides in Prostate Cancer DU-145 Cells

**DOI:** 10.3390/md16040125

**Published:** 2018-04-12

**Authors:** Zong-Ze Wu, Guo-Fang Ding, Fang-Fang Huang, Zui-Su Yang, Fang-Miao Yu, Yun-Ping Tang, Ying-Lu Jia, Yuan-Yuan Zheng, Rui Chen

**Affiliations:** 1 School of Food Science and Pharmacy of Zhejiang Ocean University, Zhejiang Provincial Key Engineering Technology Research Center of Biomedical Products, Zhoushan 316022, China; zongze461@sina.com (Z.-Z.W.); abc1967@126.com (Z.-S.Y.); ymyu@zjou.edu.cn (F.-M.Y.); tangyunping1985@163.com (Y.-P.T.); m18768013696@163.com (Y.-L.J.); zhengxiaohuo1990@163.com (Y.-Y.Z.); 15595676203@163.com (R.C.); 2 Zhejiang Fisheries Research Laboratory, Zhoushan 316021, China

**Keywords:** *Anthopleura anjunae*, morphology, mitochondria membrane potential, apoptosis rate, apoptosis-associated proteins

## Abstract

*Anthopleura anjunae* anti-tumor peptide (AAP-H) is a pentapeptide from the sea anemone *Anthopleura anjunae* with an amino acid sequence of Tyr-Val-Pro-Gly-Pro that is obtained by alkaline protease enzymatic hydrolysis extraction. In this study, we investigated the inhibitory effects of AAP-H on prostate cancer DU-145 cell proliferation using a methylthiazolyldiphenyl-tetrazolium bromide assay. Cell morphology was analyzed by hematoxylin-eosin staining, acridine orange/ethidium bromide fluorescence staining, Hoechst 33258 fluorescence staining, and scanning electron microscopy. The mitochondrial membrane potential was determined by flow cytometry following JC-1 staining. The cell apoptosis rate was measured by Annexin V-fluorescein isothiocyanate and propidium iodide staining followed by flow cytometric analysis, and the expression of apoptosis-associated proteins was assayed by Western blotting. The results demonstrated that AAP-H induced significant reductions in the number of viable cells and increased cell death in both a dose-dependent and time-dependent manner, with an IC_50_ of approximately 9.605 mM, 7.910 mM, and 2.298 mM at 24 h, 48 h, and 72 h, respectively. The morphologic characteristics of apoptotic cells were observed after treatment with AAP-H. The mitochondrial membrane potential was markedly decreased, and apoptosis increased after AAP-H treatment. Pro-apoptotic proteins, such as Bax, cytochrome-C, caspase-3, and caspase-9 were increased, but Bcl-2 was decreased. These findings suggest that AAP-H has moderate inhibitory effects on prostate cancer DU-145 cells, and the mechanism might involve the mitochondria-mediated apoptotic pathway. Therefore, AAP-H is a candidate anti-prostate cancer drug or health-care food.

## 1. Introduction

The importance of marine organisms as a source of new drug development is increasingly recognized. Marine organisms comprise more than a half of the living organisms on earth, thus providing a rich resource for unknown compounds. Several newly discovered peptides from marine organisms have been widely applied to clinical research [1,2]. Compared with terrestrial creatures, marine organisms offer enormous potential for the discovery of novel compounds due to their high-pressure, high-salt living environment, and ability to withstand great temperature differentials [3,4]. Bioactive oligopeptides extracted from various sources by enzymatic hydrolysis are used as functional food ingredients and for developing new drugs to treat or prevent various diseases due to their beneficial functions, including nerve-regulating, antihypertensive, antioxidant, antimicrobial, immunomodulatory, anti-AIDS, and anti-tumor activities [5]. A variety of biologically active peptides have been extracted from marine organisms. These active substances, which contain approximately 2–20 amino acids, exhibit remarkable biologic activities [6]. Moreover, a wide variety of mechanisms through which marine bioactive peptides induce cell death have been identified, including apoptosis, effects on the tubulin-microtubule equilibrium, and inhibition of angiogenesis. These newly discovered compounds provide clues for the application of marine peptides as lead compounds in biomedical research [7].

Since 1970, significant progress has been made in studies of sea anemones [8]. Peptide toxins produced by sea anemones exert inhibitory effects on various cancer cell lines [9,10], such as A549 lung cancer, T47 breast cancer, and A431 skin cancer [11] cells; human glioblastoma cells [12]; THP-1, JB6P+Cl41, MDA-MB-231, HeLa, and SNU-C4 cells [13]; and non-small-cell lung cancer cells [14]; and induce cell cycle arrest and apoptosis in breast cancer T47D and MCF7 cell lines [15].

Few studies have reported sea anemone muscle protein extraction methods and biologic activity. In our preliminary research, we found that a protein hydrolysate from *Anthopleura anjunae* muscle, with the sequence of Tyr-Val-Pro-Gly-Pro (AAP-H), exhibits effective antitumor activity on the prostate carcinoma DU-145 cell line. According to Harnedy and FitzGerald [16], small molecule peptides have stronger biologic activity than single amino acids, proteins, and polypeptides; therefore, small molecule peptides have advantages for medical research and health products. The biologic activity of peptides is mainly affected by the quantity and variety of their amino acids. In particular, amino acids such as Trp, Tyr, Met, Gly, Cys, His, and Pro in a peptide can significantly increase the bioactivity of the peptide [16]. Oligopeptides containing Tyr, Val, and Pro exhibit improved biologic activity. Peptides containing hydrophobic acid residues (such as Val) are better able to form oil-water interfaces, facilitating the removal of free radicals from the lipid phase [17]. In the present study, we investigated the potential anti-tumor mechanisms of AAP-H.

## 2. Results

### 2.1. Effect of AAP-H on Cell Proliferation

**Figure 1:**
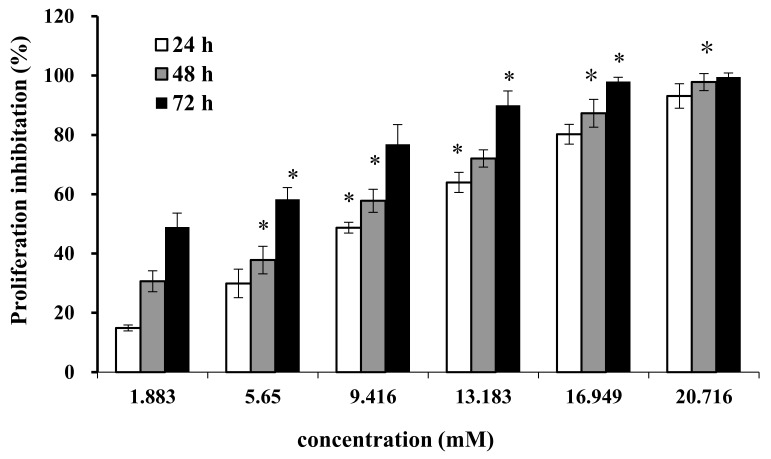
Effect of AAP-H on the growth of DU-145 cells was measured with the MTT method. Data are shown as means ± SD (*n* = 3) of three independent experiments. *p < 0.05 vs. control.

Cell proliferation and regeneration are essential for an organism to sustain growth. Abnormal cell proliferation, however, might lead to cancer or other serious diseases. Therefore, inhibition of cell proliferation is effective for tumor therapy. We treated DU-145 cells with AAP-H at different concentrations (1.883, 5.650, 9.416, 13.183, 16.949, and 20.716 mM) for 24 h, 48 h, and 72 h. AAP-H inhibited cell proliferation and induced apoptosis of DU-145 cells in a dose-dependent and time-dependent manner (Figure 1). The concentration that inhibited growth by 50% (IC_50_) at 24 h, 48 h, and 72 h was approximately 9.605 mM, 7.910 mM, and 2.298 mM, respectively.

### 2.2. Effect of AAP-H on Cell Proliferation

**Figure 2:**
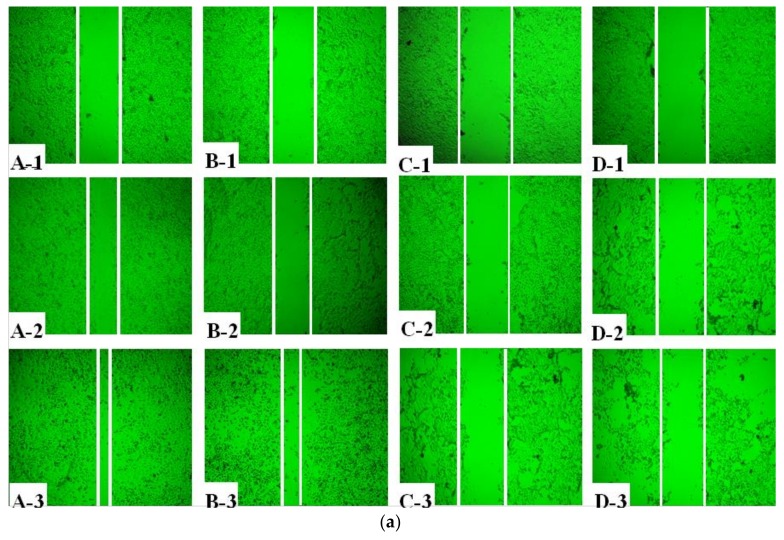

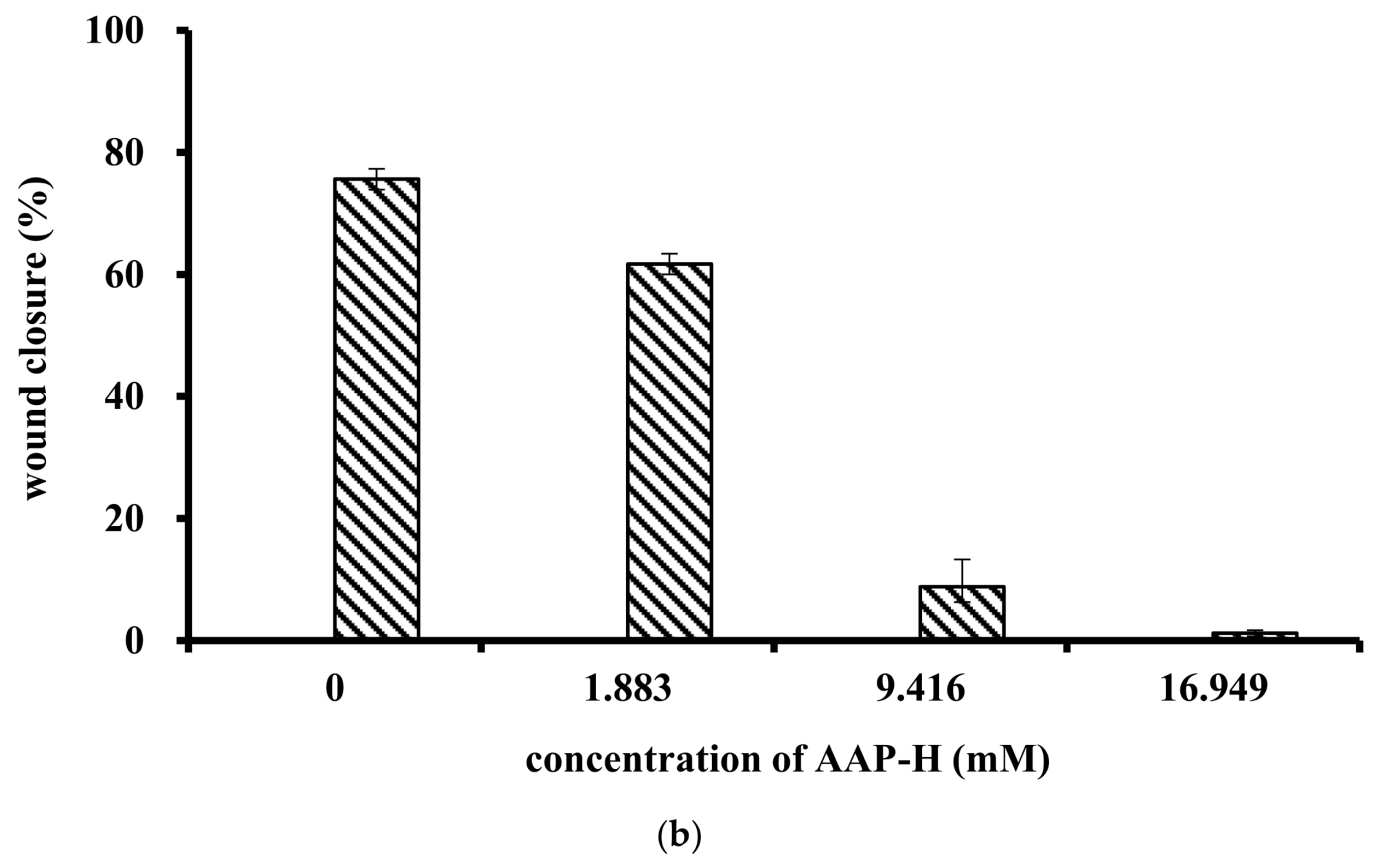
Treatment of DU-145 cells with different concentrations of AAP-H inhibited cell migration in vitro. A, B, C, and D represent cells treated with AAP-H at a concentration of 0, 1.883, 9.416, and 16.949 mM; 1, 2, and 3 represent cell migration at 0, 12, and 24 h. (**a**): The difference of cell migration with the treatment of AAP-H; (**b**): The difference of the wound healing ratio of DU-145 with the treatment of AAP-H. Magnification: ×100.

The effect of AAP-H on cell migration was determined using a wound healing assay. AAP-H inhibited DU-145 cell migration in vitro (Figure 2a). A, B, C, and D represent treatment with AAP-H at a concentration of 0, 1.883, 9.416, and 16.949 mM, respectively; and 1, 2, and 3 represent cell migration at 0 h, 12 h, and 24 h, respectively. The wound in the control group healed better than that in the AAP-H–treated group. The wound healing ratio at 12 h and 24 h (Figure 2b) indicated that AAP-H significantly inhibited wound healing by inhibiting migration of DU-145 cells.

### 2.3. Effect of AAP-H on DU-145 Cell Morphology

**Figure 3:**
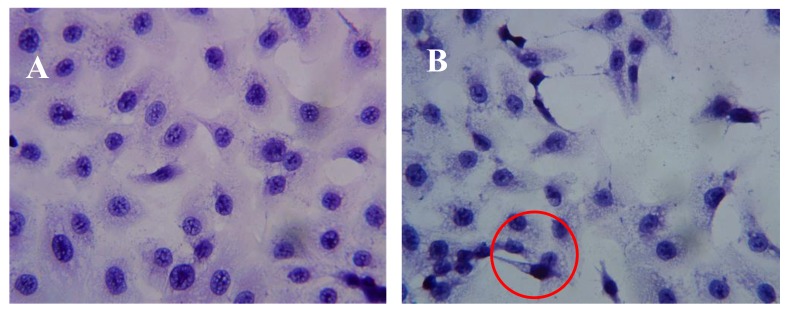

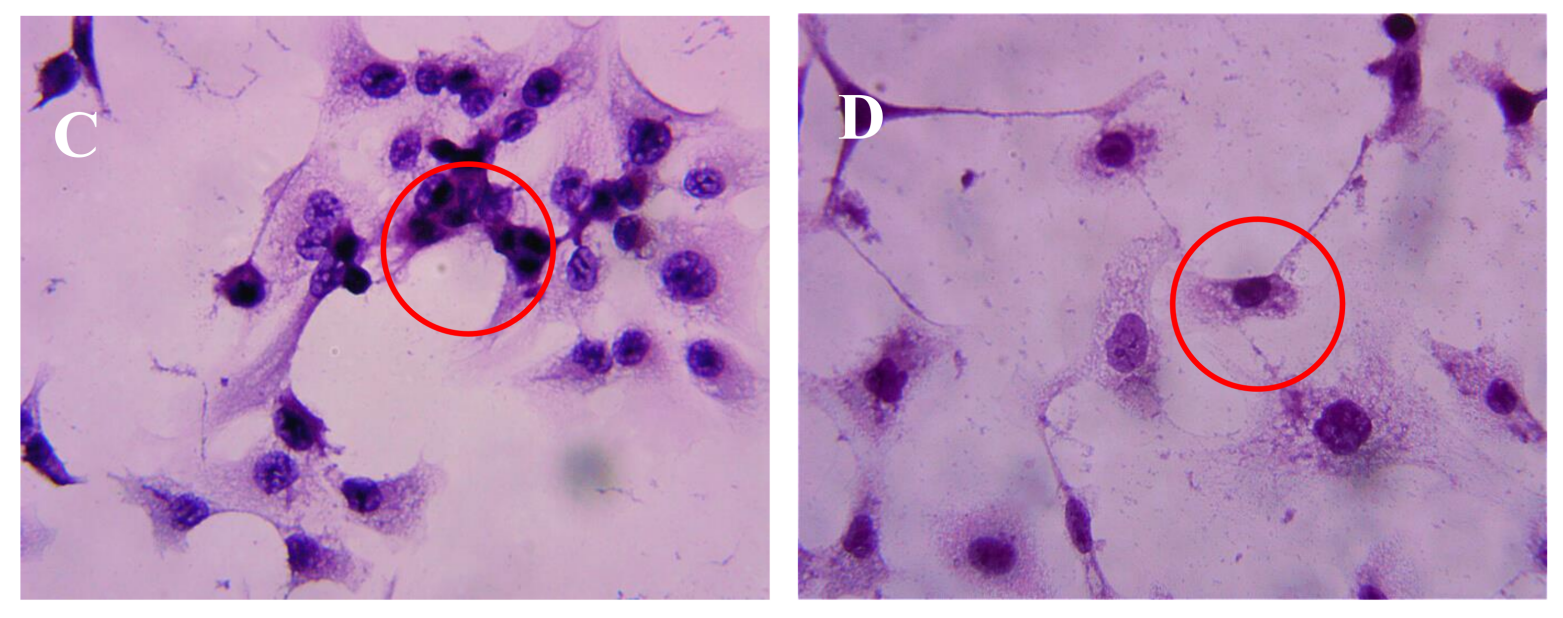
Morphologic changes of prostate cancer DU-145 cells caused by AAP-H incubation and photographed with a phase contrast microscope. (**A**) Control group; (**B**) 1.883 mM AAP-H; (**C**) 9.416 mM AAP-H; (**D**) 16.949 mM AAP-H. Magnification: ×400.

After incubation of DU-145 cells with AAP-H for various amounts of time, the cells were stained with hematoxylin and eosin (HE). The DU-145 cells in the control group showed normal membrane integrity and control group nucleus morphology (Figure 3A). DU-145 cells incubated with 1.883 mM AAP-H exhibited abnormal cell morphology, dilated intercellular spaces, and cellular shrinkage (Figure 3B). Increasing the concentration of AAP-H led to progressive changes in the cell morphology in a dose-dependent manner; the cells became shrunken, did not adhere well, floated, and clustered together (Figure 3C). Finally, in the high AAP-H concentration (16.949 mM) group, the number of cells decreased and became spindle-shaped with tentacles (Figure 3D). These morphologic changes were commonly observed during cell death induced by AAP-H.

### 2.4. Effects of AAP-H on Early- and Late-Stage Apoptosis

**Figure 4:**
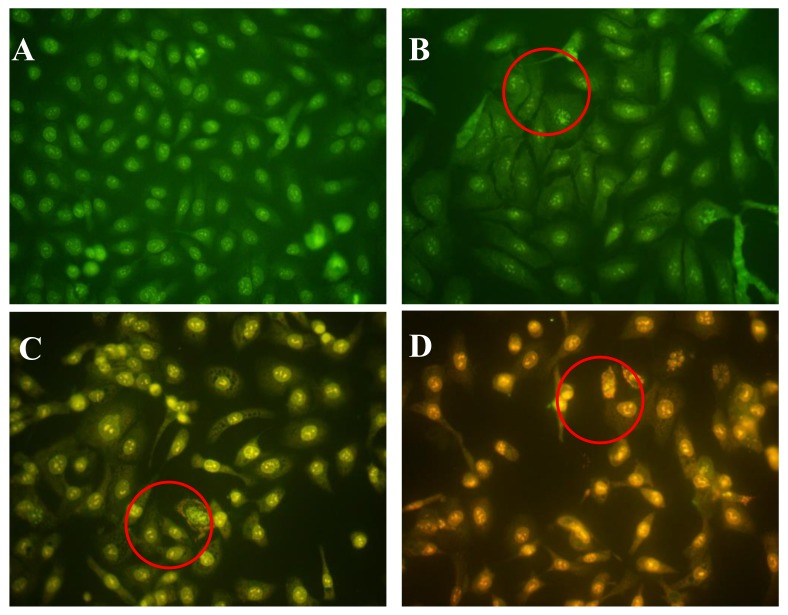
(**A**) Control group; (**B**) 1.883 mM AAP-H; (**C**) 9.416 mM AAP-H; (**D**) 16.949 mM AAP-H. Magnification: ×400.

Acridine orange/ethidium bromide (AO/EB) fluorescence staining was used to identify apoptosis-associated changes in cellular morphology during apoptosis. The method can also accurately distinguish cells at different stages of apoptosis [18]. According to instructions of the AO/EB staining kit, AO passes through live cell membranes and intercalates into DNA, which is indicated in green; EB can only pass through damaged cell membranes, indicated in orange. In control cells, live cells were stained only with AO and no significant apoptosis was detected (Figure 4A). In AAP-H–treated cells, the intercellular spaces were dilated or exhibited some small vesicles (Figure 4B), and staining was localized asymmetrically within the cells. With an increasing concentration of AAP-H, the number of early-stage apoptotic cells increased (Figure 4C). Late-stage apoptotic cells with concentrated and asymmetrically localized orange nuclear EB staining were also detected. Necrotic cells increased in volume and showed uneven orange-red fluorescence at their periphery (Figure 4D). The cells appeared to be in the process of disintegrating.

### 2.5. Effects of AAP-H on DU-145 Cell Nuclei

**Figure 5:**
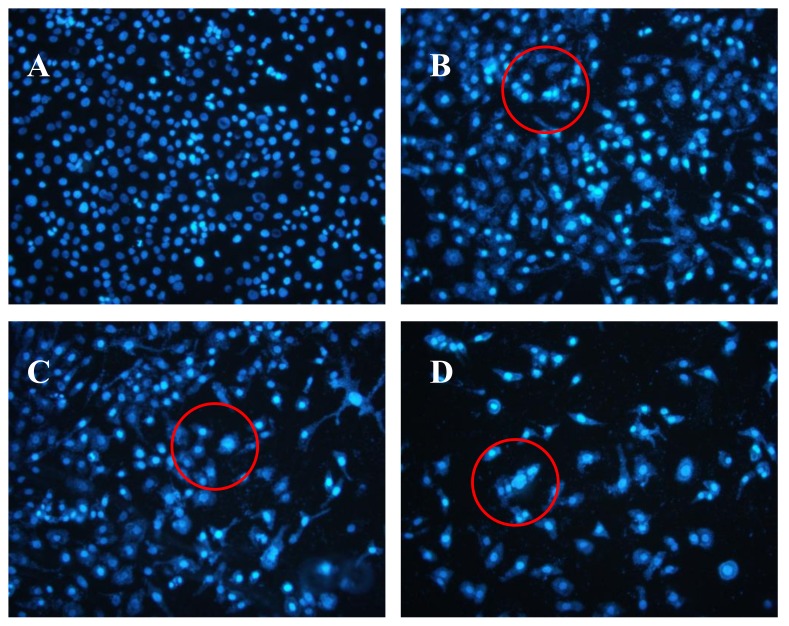
(**A**) Control group; (**B**) 1.883 mM AAP-H; (**C**) 9.416 mM AAP-H; (**D**) 16.949 mM AAP-H. Magnification: ×200.

The Hoechst 33258 fluorescent dye results are shown in Figure 5. The control group nuclei exhibited a light blue fluorescence. After treatment with AAP-H, there were obvious changes in the morphology of the nuclei. Many nuclei were pyknotic and exhibited karyorrhexis and karyolysis after the cells were treated with 1.883 mM AAP-H. In cells treated with 9.416 mM AAP-H, chromatinorrhexis was observed and the nuclear content was dispersed. Cells treated with 16.949 mM AAP-H rapidly became sparser and the nuclear chromatin in the outer nuclear layer accumulated toward the center with an uneven distribution. Typical apoptosis characteristics were observed under a transmission electron microscope.

### 2.6. Scanning Electron Microscopy Results

**Figure 6:**
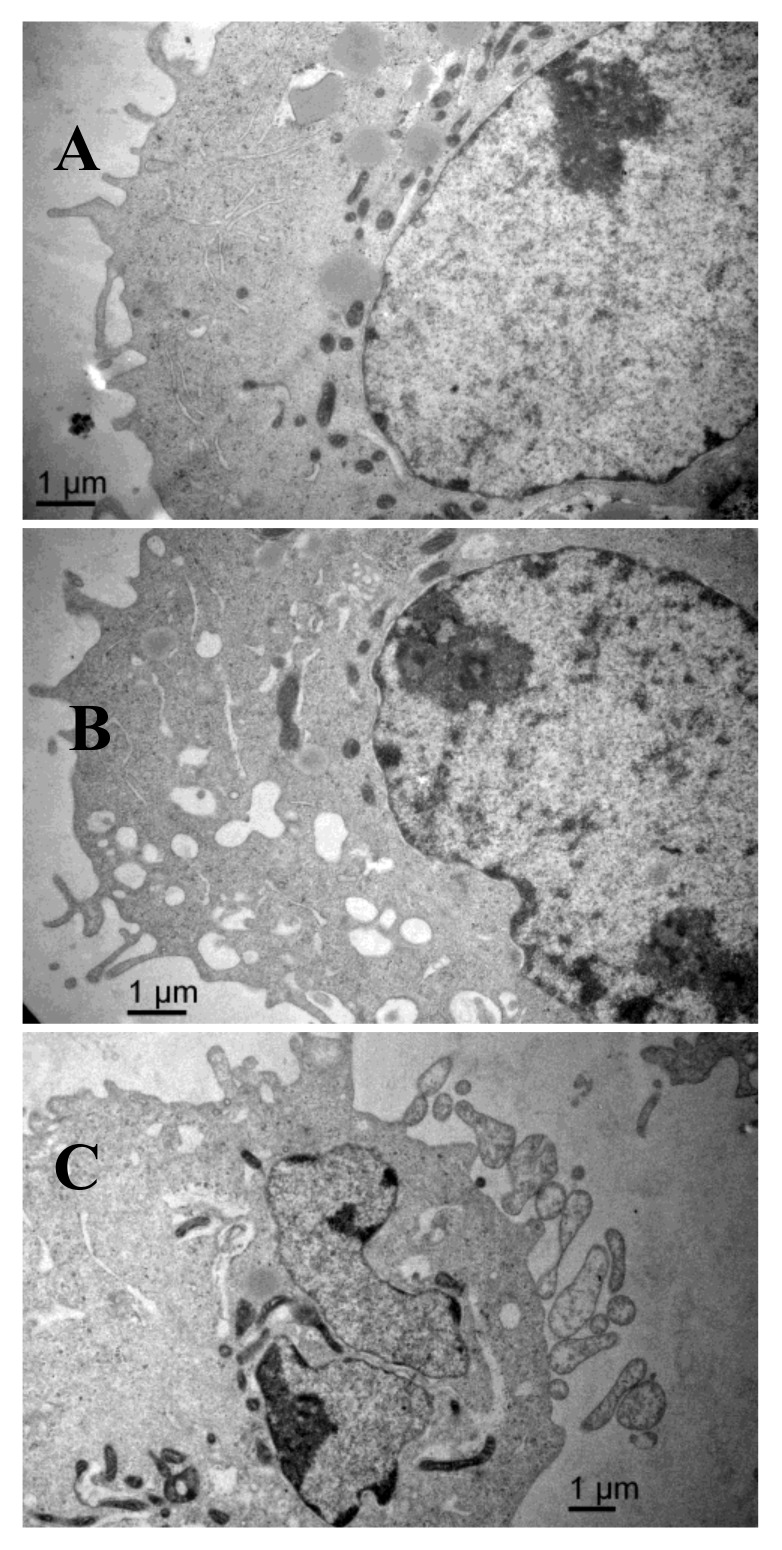
(**A**) The shape of the DU-145 cells of the control group is regular and there are plenty of microvilli on the surface, with large nuclei and prominent nucleoli, abundant mitochondria, healthy endoplasmic reticulum, and no signs of pathology in the cytoplasm. Magnification: ×15,000; (**B**) Compared with the control cells, DU-145 cells after treatment with 1.883 mM AAP-H showed typical apoptotic changes in morphology, e.g., loss of microvilli and increased vacuoles in the cytoplasm. Magnification: ×15,000; (**C**) DU-145 cells treated with 9.416 mM AAP-H exhibited disappearance of the outer microvilli and had apoptotic features: e.g., intact cell membrane, chromatin condensation, nucleic fragmentation, and apoptotic body formation. Magnification: ×15,000

The scanning electron microscopy results are shown in Figure 6. The surface of the control group cells was rich in microvilli-like structures, with cytomembrane and nuclear membrane integrity, a high amount of nuclear cytoplasm but a small amount of heterochromatin, equal size and clear nucleolus, abundant mitochondria, and endoplasmic reticulum with high-integrity cristae. After treatment with AAP-H, some apoptotic signs were visible. The outer microvilli-like structures disappeared and were replaced with lots of bubbles and apoptotic bodies in the membrane, swollen smooth endoplasmic reticulum, reduced rough endoplasmic reticulum, mitochondrial swelling, disappearing cristae, breakup of the nuclear membrane, and pyknosis of the nucleolus. These phenomena became more severe with increasing concentrations of AAP-H.

### 2.7. Effects of AAP-H on Mitochondrial Membrane Potential (Δψ_m_) in DU-145 Cancer Cells

**Figure 7:**
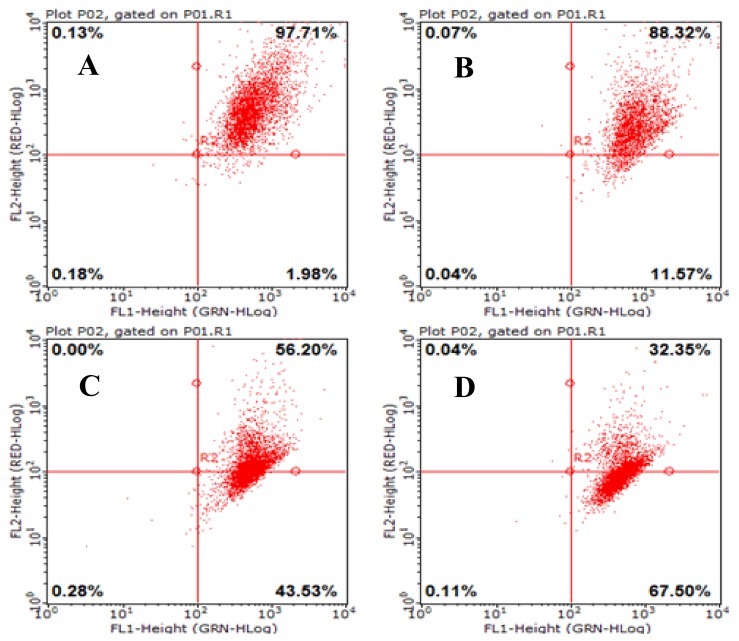
Loss of mitochondrial membrane potential was examined by JC-1 dye using flow cytometry after 24 h treatment with AAP-H. (**A**) Control group; (**B**) 1.883 mM AAP-H; (**C**) 9.416 mM AAP-H; (**D**) AAP-H, 16.949 mM AAP-H.

Mitochondria are important cell organelles, and the stability of the mitochondrial membrane potential (Δψ_m_) is critical for maintaining normal physiologic function of the cell [19]. A reduction in the Δψ_m_ was indicated by a decrease in red/green fluorescence intensity ratio. The dot plots represent the population of mitochondrial membrane polarized cells on the lower right (Figure 7, LR) and mitochondrial membrane polarized cells on the upper right (Figure 7, UR) [20]. AAP-H significantly depolarized the mitochondrial membrane potential of the DU-145 cells after 24 h incubation (Figure 7). The percentage of cells with reduced Δψ_m_ was 11.57% (1.883 mM AAP-H), 43.53% (9.416 mM AAP-H), and 67.50% (16.949 mM AAP-H). The results showed that Δψ_m_ decreased in a dose-dependent manner after 24 h treatment with AAP-H.

### 2.8. AAP-H Induces Apoptosis of DU-145 Cells

**Figure 8:**
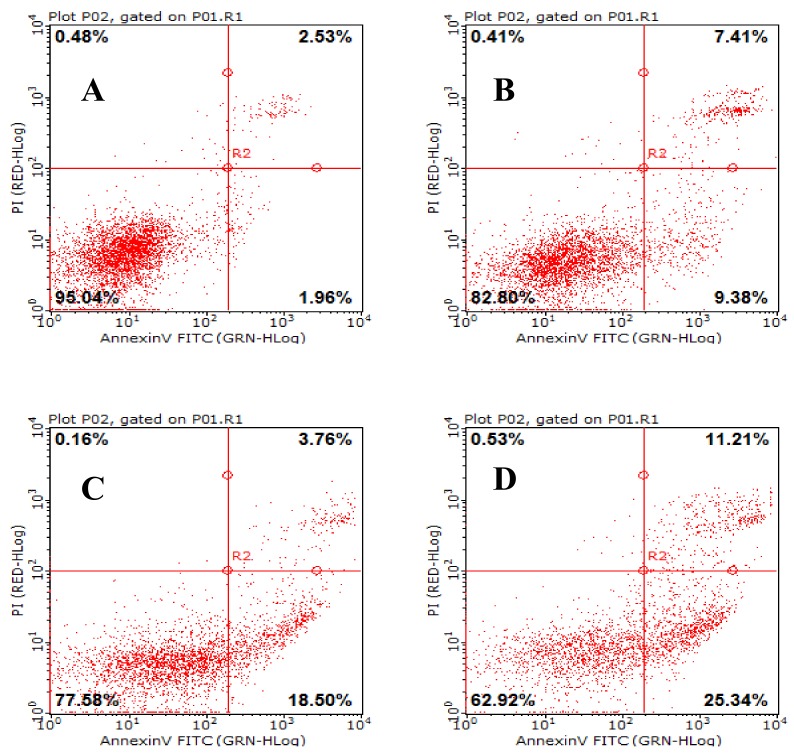
The effect of AAP-H on apoptosis in DU-145 cells as determined by Annexin V-FITC/PI Apoptosis Detection Kit staining. Detection of apoptosis and necrosis in DU-145 cells by flow cytometry. (**A**) Control group; (**B**) 1.883 mM AAP-H; (**C**) 9.416 mM AAP-H; (**D**) 16.949 mM AAP-H.

Apoptosis is a general mechanism for cleaning unwanted cells from organisms and plays a protective role against carcinogenesis [21] . Evidence from both in vivo and in vitro experiments shows that apoptosis is involved in successful cancer treatments using many drugs and other chemical substances [22]. Our experiments showed that AAP-H induced apoptosis in DU-145 cells treated with 1.883, 9.416, or 16.949 mM AAP-H for 24 h. The results indicated that apoptosis was clearly induced by AAP-H in a dose-dependent manner (Figure 8). The DU-145 cells were stained with Annexin V-fluorescein isothiocyanate and propidium iodide and analyzed by flow cytometry. The apoptotic cell proportion is shown in Figure 8.

### 2.9. Effects of AAP-H on Apoptosis-Associated Protein Levels in DU-145 Cells

**Figure 9:**
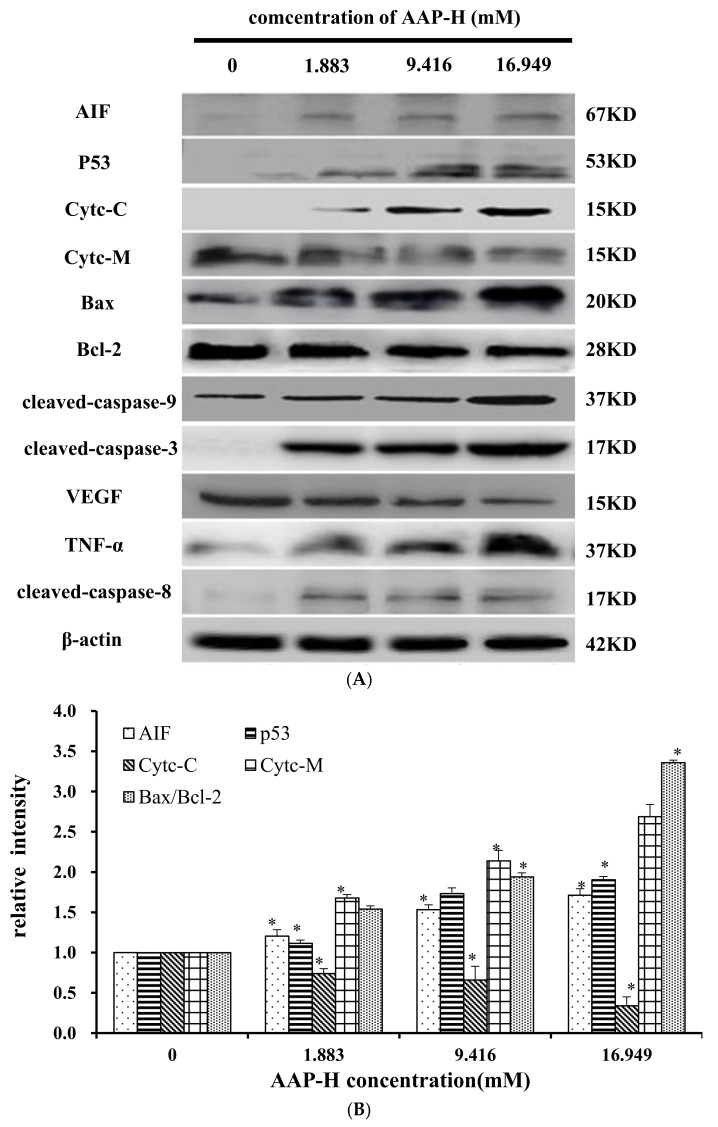

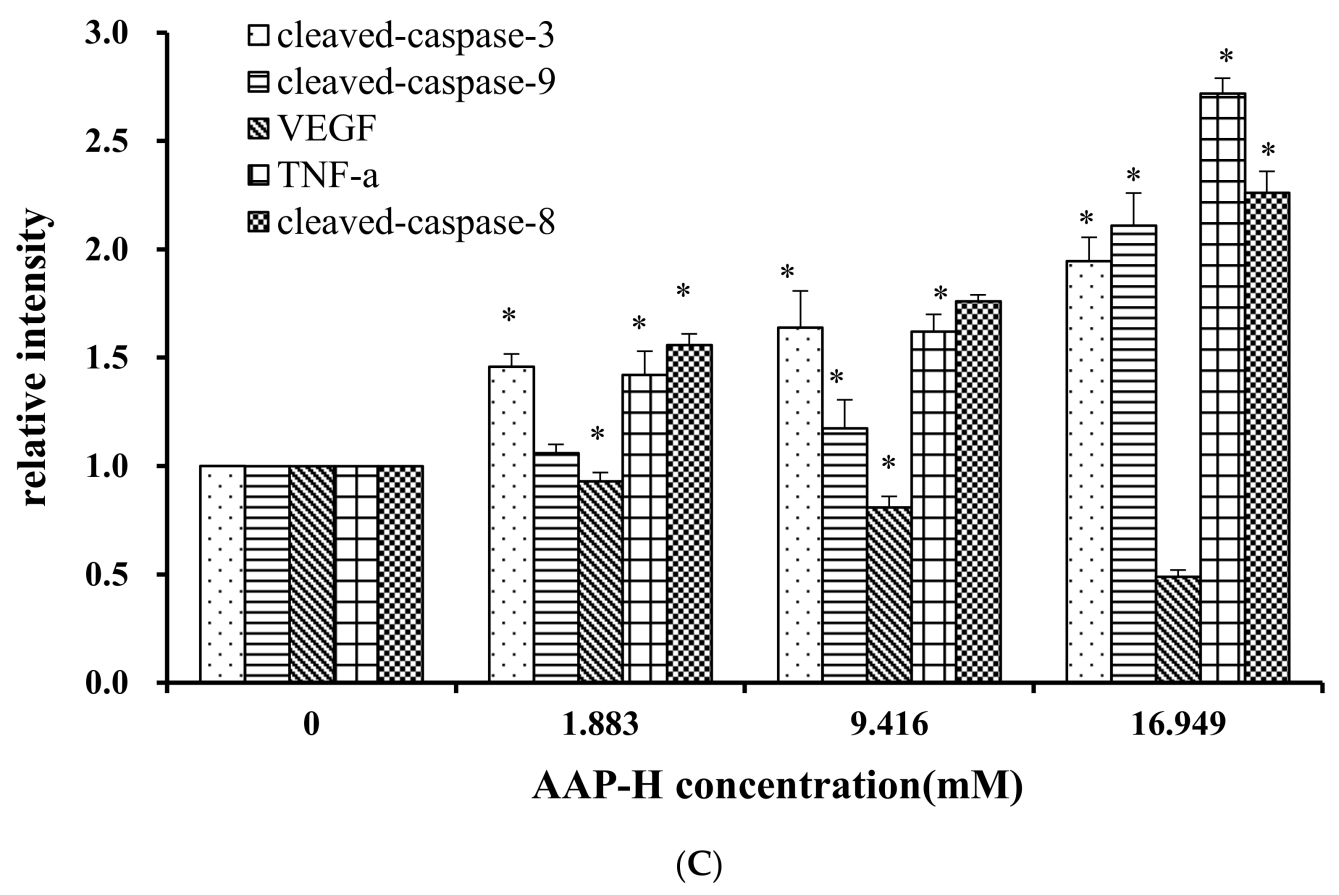
(**A**) Expression of AIF, p53, Cytc-M, Cytc-C, Bax, Bcl-2, cleaved-caspase-9, cleaved-caspase-3, VEGF, TNF-α, and cleaved-caspase-8 proteins in DU-145 cells treated with AAP-H for 24 h (**B**) Expression of AIF, p53, Cytc-c, Cytc-M, and Bax/Bcl-2 in DU-145 cells treated with AAP-H for 24 h, * *p* < 0.05 vs. control. (**C**) Expression of cleaved-caspase-3, cleaved-caspase-9, VEGF, tnf-a, cleaved-caspase-8 proteins in DU-145 cells treated with AAP-H for 24 h, * *p* < 0.05 vs. control.

The apoptosis-associated proteins induced by AAP-H were detected by Western blotting. As shown in Figure 9, the levels of apoptosis-inducing factor (AIF), p53, cytoplasm cytochrome c, Bax, cleaved caspase 9, cleaved caspase 3, tumor necrosis factor-α (TNF-α), and cleaved caspase 8 increased with an increased concentration of AAP-H, but the expression of Bcl-2, mitochondria cytochrome c, vascular endothelial growth factor (VEGF) were decreased with an increased concentration of AAP-H. Based on previous research, Bcl-2 family proteins located on the mitochondrial membrane play an indispensable role in suppressing the mitochondrial manifestations of apoptosis [23]. The mitochondrial-mediated apoptosis pathway is a process controlled by multiple genes, such as the Bcl-2 family of antiapoptotic (bcl-2, bcl-xl, mcl-1) and proapoptotic proteins (bax, bad, and bak) [24]. The Bax/Bcl-2 ratio may be more important than either promoter alone in determining apoptosis [25]. As shown in Figure 9A, AAP-H inhibited the expression of Bcl-2 but promoted the expression of Bax. As described by Harnedy and FitzGerald [16], an increased Bax/Bcl-2 ratio indicates greater vulnerability to apoptotic activation, and thus our results indicate that AAP-H interfered with the cell apoptosis signal.

These findings, especially the mitochondrial membrane potential (Δψ_m_) changes (Section 2.7), combined with previous findings, indicate that AAP-H–induced apoptosis involves the mitochondrial pathway, in which cytochrome c plays an especially important role. To test this hypothesis, we analyzed the expression of cytochrome c in mitochondria (Cytc-M) and cytochrome c in the cytoplasm (Cytc-C; Figure 9B). The expression of Cytc-M was positively related to the increase in the AAP-H concentration. Release of cytochrome c from the mitochondria into the cytosol is implicated as an important step in apoptosis. In the cytosol, cytochrome c combines with the CED-4 homologue, Apaf-1, thereby triggering Apaf-1–mediated activation of caspase-9 [19]. Intrinsic apoptosis is a mitochondria-centered cell death mediated by mitochondrial outer membrane permeabilization (MOMP) resulting in apoptosome formation, activation of caspase-9, and subsequent activation of effector caspases [26].

## 3. Discussion

In recent years, more and more oligopeptides which was obtained from marine organisms possessed anticancer activity. It has been reported that oligopeptides isolated from Bullacta exarate [27], Mytilus coruscus [28] and Sepia ink [29] so on, possessed anticancer activity. For instance, two novel oligopeptide derived from *Sinonovacula constricta*, with an N-terminal amino acid sequence identified as Leu-Pro-Gly-Pro and Asp-Tyr-Val-Pro, effectively induces apoptosis of prostate cancer cells [30]. In addition, Gln-Pro-Lys, a novel tripeptide derived from the sepia ink, has been demonstrated to exhibit anticancer properties by inducing apoptosis in Prostate Cancer cell Lines via Caspase-3 Activation and Elevation of Bax/Bcl-2 Ratio [29].

The results of the present study revealed that AAP-H inhibited DU-145 cell proliferation and is a promising candidate anti-prostate cancer agent. AAP-H is considered to suppress cancer cell growth by anti-proliferation activities and induction of apoptosis. AAP-H reduced the percentage of viable cells in a concentration- and time-dependent manner, and induced morphologic changes characterized by typical morphologic and biochemical hallmarks, including cell shrinkage, nuclear DNA fragmentation, and membrane blebbing [31]. Mitochondrial membrane potential vanishes with irrevocable cell apoptosis [31], followed by chromatin pyknosis and DNA fragmentation [32,33]. Based on the observations described in Section 2.5 and Section 2.6, treatment with AAP-H reduced the mitochondrial membrane potential and increased the apoptosis rate. These findings suggest that AAP-H plays a pivotal role in the regulation of cell proliferation through apoptosis pathways. The Western blotting results showed that AAP-H promoted increased levels of Bax, cytochrome c, caspase 9, and caspase 3, but reduced the expression of Bcl-2. These results further support the notion that AAP-H–induced apoptosis is mediated by the mitochondrial pathway. Further experiments of AAP-H will be conducted to find additional important information regarding its efficacy, toxicity and how AAP-H could be used to specifically inhibit tumor growth in vivo.

## 4. Materials and Methods

### 4.1. Reagents and Preparation of Anthopleura anjunae Anti-Tumor Peptide

The MTT Cell Proliferation and Cytotoxicity Assay kits were obtained from Beyotime Biotechnology Co., Shanghai, China; F-12 Nutrient Mixture medium was obtained from Thermo Fisher Scientific, Shanghai, China); fetal bovine serum was purchased from Sijiqing Biological Technology Co., Hangzhou, China; AO and EB were obtained from BIOSHARP China; and the Annexin V FITC PI apoptosis detection kit was purchased from Bestbio, Shanghai, China. The mitochondrial membrane potential assay kit with JC-1 was obtained from Nanjing Keygen Biotech Co., Nanjing China. Primary antibodies for β-actin, Bax, Bcl-2, cyt-c, caspase-3, caspase-8, caspase-9, TNF-α, and VEGF, and the secondary antibody were purchased from Beyotime Biotechnology Co., Shanghai, China. The enhanced chemiluminescent (ECL) detection system kit was obtained from Beyotime Biotechnology Co. All other reagents were of analytical grade. AAP-H was dissolved to the final concentrations in culture medium.

**Figure 10:**
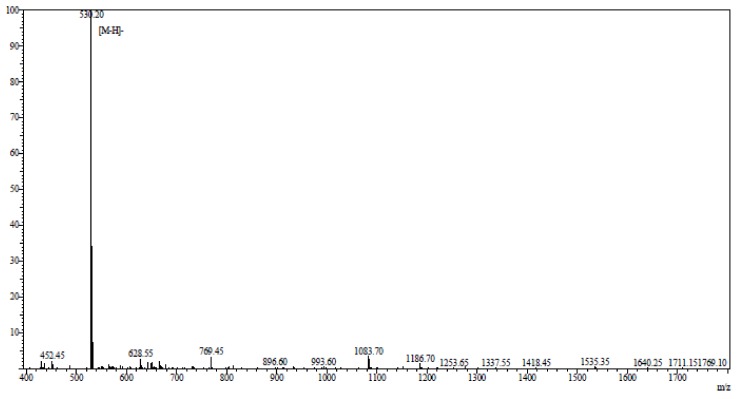
Mass spectrogram of *Anthopleura anjunae* anti-tumor peptide (AAP-H). AAP-H sequences were identified as Tyr-Val-Pro-Gly-Pro.

AAP-H was hydrolyzed by pepsin, alkaline protease, neutral protease, or trypsin. Among these, alkaline protease was the most effective. The best hydrolysis process, including temperature, time, dose, pH, and feed solution proportion, was determined by systematic testing. The hydrolysis conditions were compared to hydrolysis obtained using the protease Alcalase^®^ (2000 U/g, Millipore Sigma, Burlington, MA, USA) for 6 h at 35 °C, pH 11, and a solid-liquid ratio of 1:5. AAP-H was identified using an LTQ-Orbitrap mass spectrometer (Thermoscientific, Waltham, MA, USA) coupled with an electrospray ionization source. The molecular mass of AAP-H was determined by mass spectroscopy. AAP-H was sequenced on a Shimadzu PPSQ-31A automated gas phase sequencer (Shimadzu, Corporation, Kyoto, Japan). The purified sample was removed after centrifugation, 15 µL polybrene was added to a glass fiber disk, and dried with a stream of nitrogen, the glass fiber membrane was pretreated 5 times, and the samples were applied to the pretreated glass fiber membrane and dried with nitrogen. The sequencing results (Figure 10), published as a preliminary study revealed that the amino acid sequence of AAP-H was Tyr-Val-Pro-Gly-Pro, the molecular formula was C26H37N5O7, and the molecular mass was 531.60 Da [34].

### 4.2. Cell Culture

Cell culture was performed as described by Huang et al. [18]. The human prostate cancer DU-145 cell line was obtained from the Shanghai Cell Bank of the Chinese Academy of Sciences and cultured using standard procedures.

### 4.3. MTT Assay

The bioactivity of AAP-H was determined by MTT assay [34]. A 200-µL aliquot of DU-145 cell suspension was seeded into each well of 96-well flat-bottom plates, with an initial density of 1 × 10^5^ cells/mL, and incubated for 24 h (37 °C, 5% CO_2_). The cells were then washed three times with PBS and treated with AAP-H at final concentrations of 1.883, 5.650, 9.416, 13.183, 16.949, or 20.716 mM. The control group received just F12 (10% FBS) medium without AAP-H and was incubated in a humidified incubator (37 °C, 5% CO_2_) for 24, 48, or 72 h. Cell morphology was evaluated using an inverted microscope. The original culture medium was discarded, and then 200 µL PBS containing 10% MTT was added to each well. After a 4-h incubation, the medium was removed and 150 µL of dimethyl sulfoxide was added and the plates were placed on a TYZD-I oscillator (BI-LANG Instruments Inc., Shanghai, China) and vibrated for 15 min. The absorbance was measured at 490 nm and percentage of viable cells was calculated according to the following formula: inhibition (%) = [(OD_control_ − OD_treated_)/(OD_control_ − OD_blank_)] × 100%.

### 4.4. Morphological Analysis

#### 4.4.1. Wound Healing Assay

Four lines were drawn with a marker on the bottom of each well of 6-wells plates. To determine the effect of AAP-H on DU-145 cell migration, the cells were seeded at a density of 1 × 10^5^ cells/mL in each well and cultured at 37 °C in a 5% CO_2_ incubator until reaching 90~95% confluence. Using a sterile 200 µL pipet tip, three wounds were scratched through the cells perpendicular to one of the lines drawn with the marker. The cells were rinsed with PBS, and various concentrations of AAP-H (0, 1.883, 9.416, and 16.949 mM) were added in culture medium, and the cells were cultured at 37 °C in a 5% CO_2_ incubator. Photomicrographs were taken using a phase contrast microscope with a 10 × lens. Photomicrographs were taken just above and below each line, and pictures were obtained at 0, 12, and 24 h. The wound area was measured with Image software. Relative mobility was calculated for 12 and 24 h after the wound: relative mobility (%) = (Wound Area (t_0_) − Wound Area (t_24_)/Wound Area (t_0_)) × 100.

#### 4.4.2. Hematoxylin-Eosin Staining

Cell culture was performed as described by Huang et al. [18]. The prostate carcinoma DU-145 cells were suspended at a final concentration of 1 × 10^5^ cells/mL and cultured in a -well flat-bottomed microplate over a 20 × 24 mm coverslip and cultured for 24 h. Experimental groups were exposed to 1.883, 9.416, or 16.949 mM doses of AAP-H for 24 h, and the negative control was not treated with AAP-H. After 24 h, the morphological changes were evaluated using an inverted microscope. The cells were then washed with PBS, fixed in 95% alcohol, stained with hematoxylin-eosin, dehydrated in an ascending series of ethanol, and defatted in dimethylbenzene.

#### 4.4.3. Morphological Analysis by Acridine Orange/Ethidium Bromide Fluorescent Staining

Cells morphology was evaluated using AO/EB fluorescence staining as described by Liu et al. [17]. To accurately distinguish cells at different stages of apoptosis, the prostate carcinoma DU-145 cells in logarithmic growth phase were digested with 0.25% trypsin and suspended at a final concentration of 1 × 10^5^ cells/mL, cultured at 37 °C in a 5% CO_2_ incubator for the indicated time and stained with AO/EB. Apoptotic cell morphology was examined using a fluorescence microscope (OLYMPUS, Tokyo, Japan). This staining assay was repeated 3 times.

#### 4.4.4. Hoechst 33258 Fluorescent Staining

Cells were seeded in 25-ml culture vessel to 80% confluence, and various concentrations of AAP-H (1.883, 9.416, or 16.949 mM) in culture medium was added. The control group was treated with the same volume of F-12 culture medium without AAP-H and then cultured at 37 °C in a 5% CO_2_ incubator for the indicated time and stained with Hoechst 33258.

#### 4.4.5. Scanning Electron Microscopy

Cells were seeded in 25-mL culture dishes to 80% confluence, AAP-H (0, 1.883, and 9.416 mM) in F-12 culture medium was added, and then cultured at 37 °C in a 5% CO_2_ incubator for 24 h. The cells were collected and immobilized according to standard protocols, and photographs were obtained using a scanning electron microscope.

### 4.5. Mitochondrial Membrane Potential Change Effected by AAP-H

Cells were seeded in 25-mL culture dishes at a density of 1 × 10^5^ cells/mL and allowed to adhere overnight. Experimental groups were exposed to 1.883, 9.416, or 16.949 mM AAP-H for 24 h, and the negative control was treated with only F-12 culture medium. After 24 h, the cells were washed with PBS and evaluated according to the instructions for the mitochondrial membrane potential assay kit (JC-1) (Nanjing Keygen Biotech Co., Nanjing, China). Changes in mitochondrial membrane potential were analyzed by flow cytometry.

### 4.6. AAP-H Induced Apoptosis

Apoptosis of DU-145 cells induced by AAP-H was confirmed according to Huang et al. [18]. Early and late apoptosis were measured by Annexin V-FITC and propidium iodide (PI) double-staining and detected using a Guava^®^ easyCyte HT Flow Cytometer. Cells were incubated as described in Section 4.5, and after a prescribed incubation time, cells were digested with 0.25% trypsin, washed with PBS, and centrifuged at 1000 rpm at 4 °C for 5 min. The cells were resuspended in 400 µL of 1× Annexin V binding buffer. Next, 5 µL Annexin V-FITC was added and the cells were incubated at room temperature in the dark, and after 15 min 5 µL PI was added and the cells were incubated further at 2–8 °C for 5 min in the dark. The apoptosis rate was analyzed by flow cytometry.

### 4.7. Western Blotting Analysis of Specific Proteins

Western blotting was performed as described by Ding et al. [32]. Total proteins were extracted with RIPA buffer containing phenylmethylsulfonyl fluoride and the concentration was determined using a BCA Protein Assay Kit. Mitochondrial and cytoplasmic cytochrome C were extracted using a mitochondria isolation kit. Apoptosis-related proteins were separated by sodium dodecyl sulfate-polyacrylamide gel electrophoresis and blotted onto polyvinylidene difluoride (PVDF) membranes. The membrane was blocked with 5% powdered non-fat milk in 0.1% Tween 20 in PBS for 1 h at room temperature on a shaking-table, and then incubated with diluted primary antibodies overnight at 4 °C. The PVDF membranes were then incubated with the corresponding secondary antibody for 2 h at room temperature. After washing twice with Tris-buffered saline with 0.1% Tween20 and once with Tris-buffered saline, the PVDF membranes were stained with ECL reagents, exposed to Fluor Chem FC3 chemical luminescence imaging system in accordance with the manufacturer’s instructions, and target protein expression was quantified by densitometry as shown in DRAFT-alphaview.

### 4.8. Statistical Analysis

The data were analyzed using SPSS software (version 19.0) and are presented as the mean ± SD (*n* = 3). Differences were considered significant when the p value was less than 0.05.

## 5. Conclusions

In conclusion, the results of the present study demonstrated that AAP-H is cytotoxic to prostate cancer DU-145 cells through the induction of apoptosis via mitochondrial pathways and death receptor pathways. These findings provide possible molecular mechanisms of the anti-prostate cancer activity of AAP-H and confirm the potential of AAP-H as an anti-prostate cancer drug candidate.
